# Individual Variations in Inorganic Arsenic Metabolism Associated with *AS3MT* Genetic Polymorphisms

**DOI:** 10.3390/ijms12042351

**Published:** 2011-04-04

**Authors:** Tetsuro Agusa, Junko Fujihara, Haruo Takeshita, Hisato Iwata

**Affiliations:** 1 Department of Legal Medicine, Faculty of Medicine, Shimane University, Enya 89–1, Izumo 693–8501, Japan; E-Mails: ax@med.shimane-u.ac.jp or ax@agr.ehime-u.ac.jp (T.A.); jfujihara@med.shimane-u.ac.jp (J.F.); htakeshi@med.shimane-u.ac.jp (H.T.); 2 Center for Marine Environmental Studies (CMES), Ehime University, Bunkyo-cho 2–5, Matsuyama 790–8577, Japan

**Keywords:** arsenic, *AS3MT*, genetic polymorphism

## Abstract

Individual variations in inorganic arsenic metabolism may influence the toxic effects. Arsenic (+3 oxidation state) methyltransferase (AS3MT) that can catalyze the transfer of a methyl group from *S*-adenosyl-l-methionine (AdoMet) to trivalent arsenical, may play a role in arsenic metabolism in humans. Since the genetic polymorphisms of *AS3MT* gene may be associated with the susceptibility to inorganic arsenic toxicity, relationships of several single nucleotide polymorphisms (SNPs) in *AS3MT* with inorganic arsenic metabolism have been investigated. Here, we summarize our recent findings and other previous studies on the inorganic arsenic metabolism and *AS3MT* genetic polymorphisms in humans. Results of genotype dependent differences in arsenic metabolism for most of SNPs in *AS3MT* were Inconsistent throughout the studies. Nevertheless, two SNPs, *AS3MT* 12390 (rs3740393) and 14458 (rs11191439) were consistently related to arsenic methylation regardless of the populations examined for the analysis. Thus, these SNPs may be useful indicators to predict the arsenic metabolism via methylation pathways.

## 1. Introduction

Groundwater pollution by inorganic arsenic (IA) derived from natural origin is a serious issue of public health in the world, especially in developing countries where clean and safe surface water is not available in many places. In these areas, high arsenic concentrations exceeding the WHO guideline (10 μg/L) for drinking [1] have been reported in the groundwater [2–4]. It is well known that IA is one of the carcinogenic chemicals for the human. Chronic arsenic exposure causes skin pigmentation, hyperkeratosis, and cancers in the skin, lung, bladder, liver, and kidney resulting in high mortalities [1,5,6].

In general, humans can metabolize ingested IA to monomethylated arsenic (MMA) and then dimethylated arsenic (DMA), through arsenic (+3 oxidation state) methyltransferase (AS3MT). The pathways are discussed in detail in a later section of this review. Urinary arsenical profile has been used to assess arsenic exposure and its metabolism in individuals. In general, concentration ratios of MMA/IA and DMA/MMA in the urine indicate first and second methylation capacity, respectively. The metabolic action of AS3MT is considered to have an association with accumulation profile of various arsenic compounds in the body and consequently with susceptibility to toxic effects of arsenic. Several previous epidemiological studies showed that people who had high percentage of DMA (%DMA) in the urine could excrete more arsenic from the body because of high arsenic methylation capacity [7,8]. In other epidemiological studies, high concentration or percentage of MMA in the urine of populations with arsenic-related diseases were reported when compared with that of healthy people [9–12].

Vahter [13] showed that relative distributions of IA, MMA, and DMA in the urine of various populations are generally 10–30%, 10–20%, and 60–70%, respectively. On the other hand, there are large variations in the arsenic metabolism at individual and population levels [7]. It is known that biological and environmental factors including age, sex, pregnancy, arsenic exposure level, smoking habits, nutritional status and diet, potentially relate to the inter-individual variation (reviewed by Tseng [14]). With regard to ethnic related variation, it was shown that Andes women in arsenic contaminated area had only a low percentage of MMA[V] (0–11%) in the urine [15], whereas residents ingesting arsenic through drinking water in the northeast Taiwan had an extraordinarily high %MMA[V] (26.9%) in the urine [16]. These results indicate that genetic polymorphisms of certain enzymes that can metabolize arsenic through methylation pathways may be one of factors associated with the variation of arsenic profiles.

Genetic analyses of SNPs in the enzymes that may be involved in arsenic metabolism have been recently developed to evaluate the variation in the metabolism or toxic impacts of arsenic at individual or population level. We have also reported the association of arsenic metabolism-SNPs in *AS3MT* in Vietnamese [17–21]. However, a limited number of publications regarding the association of SNPs in *AS3MT* with arsenic methlyation are available. Moreover, the published results are based on fragmentary investigations and thus consistent conclusions have not yet been drawn. Hernandez and Marcos [22] have recently reviewed the role of genetic polymorphisms in enzymes related to arsenic metabolism including AS3MT. Thereafter, the number of publications regarding genetic polymorphisms dependent metabolism and toxicity of arsenic is increasing.

In this short-review, we address the following three topics: (1) enzymatic processes of arsenic methylation by AS3MT; (2) associations of genetic polymorphisms in *AS3MT* with variation in arsenic methylation; (3) frequency distributions of genetic polymorphisms in *AS3MT* at population levels. Based on the summarized data, we suggest which SNPs in *AS3MT* gene play an important role in arsenic methylation. In addition, we discuss future issues to be solved.

## 2. Arsenic Methylation by Human AS3MT

**Figure 1.**
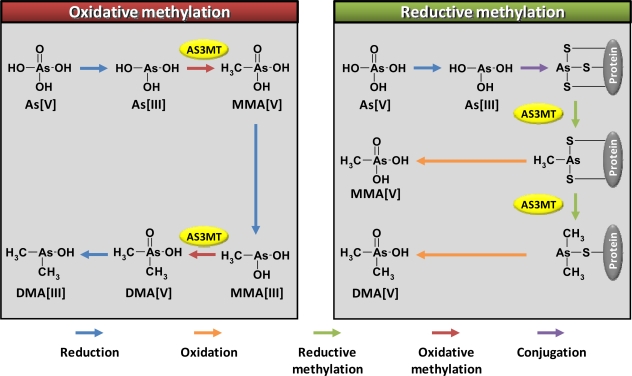
Proposed pathways of inorganic arsenic metabolism by oxidative and reductive methylation.

Two distinct pathways of IA methylation (oxidative and reductive) have been proposed (Figure 1). A model of oxidative methylation of IA was proposed by Challenger [23] and Cullen and Reimer [24]. By the combination of oxidative methylation and other pathways, arsenate (As[V]) is transformed to dimethylarsinous acid (DMA[III]); arsenate (As[V]) → arsenite (As[III]) → monomethylarsonic acid (MMA[V]) → monomethylarsonous acid (MMA[III]) → dimethylarsinic acid (DMA[V]) → dimethylarsinous acid (DMA[III]) (Figure 1). However, this sequential pathway does not fully explain the reason why the intermediate species, DMA[V] is detected as a major arsenical in the urine of humans. Methylation has been considered as a detoxification process of IA, because toxicities of MMA[V] and DMA[V] are much lower than that of IA. On the other hand, later studies revealed that MMA[III] or DMA[III] are more cytotoxic and genotoxic than IA [25,26], suggesting that oxidative methylation of IA is a bioactivation process.

On the contrary, Hayakawa *et al*. [27] reported a reductive methylation model for the metabolism of arsenic compounds. In this model, trivalent arsenicals are conjugated with glutathione (GSH) and then are methylated; As[III] → arsenotriglutathione (As[III](GS)_3_) → monomethylarsenodiglutathione (MMA[III](GS)_2_) → dimethylarsenoglutathione (DMA[III](GS)) (Figure 1). MMA[III](GS)_2_ and DMA[III](GS) are then oxidized to MMA[V] and DMA[V], respectively. Naranmadura *et al*. [28] investigated hepatic and renal metabolites of arsenic after an intravenous injection of As[III] (0.5 mg As/kg body weight) in rats. The authors proposed that trivalent arsenic species can bind to thiol groups in proteins. The protein-bound arsenicals are released from the proteins by conjugation with GSH to form As[III](GS)_3_ or MMA[III](GS)2 or DMA[III](GS) [28]. Hence, in the reductive methylation, MMA[V] and DMA[V] could be the end products. This hypothesis is consistent with the results that DMA[V] is found as a major arsenical in the urine. This sequential reaction is considered to be a detoxification process of IA, because of the low toxicities of MMA[V] and DMA[V].

Through both oxidative and reductive metabolic pathways, AS3MT plays an important role on arsenic methylation (Figure 1). Lin *et al*. [29] succeeded in purifying arsenic (III) methyltransferase (previously called as Cyt19) from the liver cytosol of adult male Fischer 344 rats. AS3MT is an S-adenosyl-L-methionine dependent enzyme and could methylate trivalent arsenicals [29,30]. Human *AS3MT* gene is composed of 32-kb with 11 exons [30]. Variety of genetic polymorphisms including SNPs and variable number of tandem repeats (VNTRs) have been identified in this gene [30,31]. This indicates that these genotypes may influence the methylation capacity of arsenic compounds and subsequently the arsenic toxicity. Recently, Kojima *et al*. [32] reported that methylation of IA by AS3MT induces oxidative DNA damage and increase their carcinogenicity *in vitro*. In the following section, we summarize *in vitro* and epidemiological studies on the AS3MT genetic polymorphism-dependent metabolism of arsenic compounds.

## 3. Association of Arsenic Methylation with Genotypes in Human *AS3MT*

**Figure 2.**
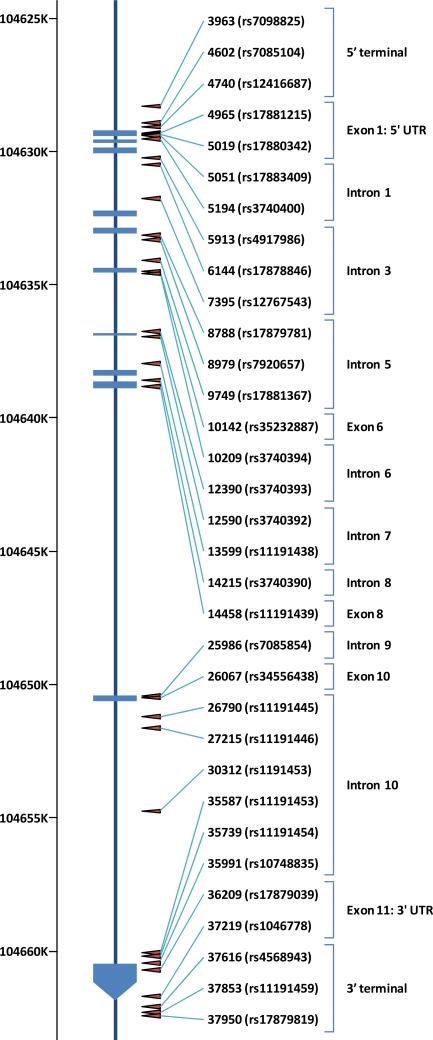
Location of genetic polymorphisms in AS3MT. Dark blue rectangles represent exons. Chromosome positions are also indicated. Arrows show the locations of genetic polymorphisms.

Notation of SNPs in human *AS3MT* is individually defined among studies. In this review, we use the notation by SNP ID which indicates the polymorphism identification number relative to the location in the consensus sequence (AY817668) with the first base of the consensus number 1 and dbSNP rs# cluster id which is available on Search for SNP on NCBI Reference Assembly [33]. Chromosome positions of genetic polymorphisms in AS3MT are indicated in Figure 2.

### 3.1. *In Vitro* Studies

Drobna *et al*. [34] reported the association of genotypes in *AS3MT* with arsenic methylation in human hepatocytes. They treated primary hepatocytes from eight human donors with graded concentrations of As[III] (0.330 nmol/mg protein) for 24 h to evaluate the variation in arsenic metabolism. One of eight donor’s cells showed a higher methylation rate than other cells at a high exposure level (30 nmol/mg protein). The methylation rate was consistent with heterozygote of Met287Thr at amino acid base (14458 at nucleotide base, rs11191439) mutation of *AS3MT*, suggesting a linkage of this genotype with arsenic methylation.

*In vitro* functional analysis of human AS3MT was performed by Wood *et al*. [30]. The authors initially sequenced DNA samples from African-American (*n* = 60) and Caucasian-American (*n* = 60), and identified 26 SNPs and one VNTR. Among the SNPs, three SNPs, Arg173Trp, Met287Thr, and Thr306Ile in the exon region were non-synonymous. In the AS3MT-expressed COS-1 cells, which was treated with 12.5 nM As[III], the enzyme activity and immunoreactive protein expression of 287Thr variant of AS3MT were significantly higher than those of the wild type, whereas 173Trp and 306Ile variants showed opposite trends. Reporter gene assays using reporter constructs containing different length 5′-UTR VNTR sequences of AS3MT gene demonstrated that the transcriptional expression of this gene depends on the number of the repeat sequence. Given these results, the authors suggested genetic polymorphisms in *AS3MT* may contribute to individual differences in the expression and function of AS3MT, and, consequently, to variation in the risk of arsenic-dependent carcinogenesis.

### 3.2. Human Case Studies

#### 3.2.1. Mexico

Case studies on human populations regarding the *AS3MT* polymorphism have recently started. Genetic association of *AS3MT* with urinary arsenic metabolite levels in residents (*n* = 135) ingesting arsenic (5.5–43.3 μg/L) through drinking water from the Yaqui Valley of Sonora, Mexico, was reported by Meza *et al*. [35]. The authors found that three unexonic SNPs (2393 at ATG offset position (SNP ID; *AS3MT* 7395, rs#; rs12767543), 7388 (*AS3MT* 12390, rs3740393), and 30585 (*AS3MT* 35587, rs11191453)) in *AS3MT* were significantly associated with DMA[V]/MMA[V] in the urine of the entire population. Subsequent analysis showed that these relationships were observed only in children (7–11 years), but not for adults (18–79 years); DMA[V]/MMA[V] for AS3MT 7395 (rs12767543) AG + AA, 12390 (rs3740393) CG + CC, and 35587 (rs11191453) CT + CC were higher than that for *AS3MT* 7395 (rs12767543) GG, 12390 (rs3740393) GG, and 35587 (rs11191453) TT, respectively. The linkage disequilibrium (LD) analysis revealed that these SNPs have significant LD, with *r^2^* = 0.56 for *AS3MT* 7395 (rs12767543) and 35587 (rs11191453), and *r^2^* = 0.94 for *AS3MT* 12390 (rs3740393) and 35587 (rs11191453). In addition, *AS3MT* 35587 (rs11191453) CT + CC had significantly higher As[III]/MMA[V] than *AS3MT* 35587 (rs11191453) TT for children.

Following the above initial study, the same group [36] found a clear association of percentage of (%) MMA[V] with genetic polymorphism in *AS3MT* 35587 (rs11191453) only for children. However, given that the participant selection may be biased; children of the above study belong to more indigenous American ancestry than adults [33,34], these age-dependent associations of genetic polymorphisms in *AS3MT* with arsenic metabolism need reconsideration.

According to Valenzuela *et al*. [37], *AS3MT* polymorphisms may influence skin lesions caused by arsenic exposure through drinking water. This group investigated three SNPs, *AS3MT* 4602 (rs7085104), 14458 (rs11191439, Met287Thr), and 35587 (rs11191453) in 71 individuals with skin lesions and 51 controls without the lesions from Zimapan, Hidalgo in Mexico. In this study area, medians of arsenic concentrations in wells and human urine samples were 84 μg/L and 109 μg/g creatinine, respectively. No significant association of *AS3MT* 35587 (rs11191439) was found with arsenic metabolism and skin disease. Among the subjects, *AS3MT* 4602 (rs7085104) GG carriers had lower %MMA[III + V], but higher %DMA[III + V] and DMA[III + V]/MMA[III + V] than 4602 (rs7085104) AA + AG carriers. The percentages of IA[V] and MMA[III + V] in *AS3MT* 14458 (rs11191439) CT + CC carriers were significantly higher than those in *AS3MT* 14458 (rs11191439) TT carriers. This suggests that *AS3MT* 14458 (rs11191439) CT + CC carriers may have a poor methylation capacity. Interestingly, the frequency of this SNP carriers in the skin lesion group was marginally (*p* = 0.055) higher than the control subjects, suggesting that *AS3MT* 14458 (rs11191439) CT + CC may be involved in skin lesions. Although the way in which genetic polymorphism of *AS3MT* 14458 (rs11191439) affects skin lesions is not clear, it can be presumed that the skin disease may be related to the metabolic pathway of IA.

Recently, from a large sample size (*n* = 405), Gomez-Rubio *et al*. [38] found significant associations of unexonic *AS3MT* 7395 (rs12767543), 12390 (rs12767543), 14215 (rs3740390), 30312 (rs10883795), 35587 (rs11191453), 35739 (rs11191454), and 37219 (rs1046778), with urinary DMA[V]/MMA[V], also showing strong LD. For example, DMA[V]/MMA[V] was higher in CC genotype of AS3MT 30312 (rs10883795), followed by TC and TT genotypes. On the contrary, exonic *AS3MT* 14458 (rs11191439) showed no significant association with arsenic methylation, probably due to the small number of variants for this SNP. As described earlier, a previous study by this group revealed a children specific association of *AS3MT* genotypes with arsenic metabolism [35,36], but the association was also observed in adults when analyses were performed using a large number of subjects [38].

Sampayo-Reyes *et al*. [39] evaluated DNA damages by a comet assay in residents who were exposed to IA from drinking water (1–187 μg/L) in Torreon, Coahuila, Mexico. The results revealed a significant positive correlation between DNA damages and urinary arsenic concentrations, suggesting that this population is under threat by exposure to arsenic. Furthermore, *AS3MT* 14458 (rs11191439, Met287Thr) influenced DNA damages in children; DNA damages of subjects having *AS3MT* 14458 (rs11191439, Met287Thr) C allele were higher than those having T allele (*p* = 0.034). Hence, the authors suggested that the arsenic exposure through drinking water induces DNA damage and this effect may become more profound by *AS3MT* 14458 (rs11191439, Met287Thr).

#### 3.2.2. Argentina

Significant relationships between genetic polymorphisms in *AS3MT* and arsenic metabolism in Argentina were reported by Schläwicke Engström *et al.* [40]. This group analyzed arsenic compounds in the urine, whole blood, and buccal cells and polymorphisms in *AS3MT* of 147 indigenous women from a village in the northern Argentinean Andes. The residents have been exposed to high levels (about 200 μg/L) of arsenic through drinking water. For *AS3MT*, nine polymorphisms (*AS3MT* 5019 (rs17880342), 5194 (rs3740400), 8788 (rs17881367), 12390 (rs3740393), 12590 (rs3740392), 14215 (rs3740390), 14458 (rs11191439), and 35991 (rs10748835)) were identified. Among these SNPs, intronic *AS3MT* 12390 (rs3740393), 14215 (rs3740390), and 35991 (rs10748835), which had a strong LD with each other (*r^2^* = 0.74–0.95), were significantly associated with the secondary methylation step of arsenic. For example, AA type of *AS3MT* 35991 (rs10748835) had a lower %MMA[V] and higher %DMA[V] and DMA[V]/MMA[V] ratios in the urine than the GG and GA types. They also found that the genotype frequencies in three SNPs in *AS3MT*, which had correlations with the secondary methylation of arsenic in a population of Argentina, were higher than those in other populations. This may be due to a positive selection of *AS3MT* genetic polymorphisms that can efficiently metabolize arsenic for detoxification, because these populations have suffered from arsenic toxicity for thousands of years [41,42].

Recently, Schläwicke Engström *et al.* [43] genotyped another two SNPs, *AS3MT* 4602 (rs7085104) in 5′ terminal and *AS3MT* 5194 (rs3740400) in intron 1, and evaluated the influences on arsenic metabolism in the same subjects as in the previous cases. These SNPs comprised a strong LD (*r^2^* = 0.97) with each other and also with *AS3MT* 12390 (rs3740393), 14215 (rs3740390), and 35991 (rs10748835) which were reported in the previous study [40]. This LD cluster was found only in this Argentinean population, but not in other HapMap populations. The SNPs, *AS3MT* 4602 (rs7085104) and 5194 (rs3740400) were clearly associated with %MMA[V] and %DMA[V].

#### 3.2.3. Chile

Hernández *et al.* [44] investigated 50 workers who have been occupationally exposed to high levels of arsenic in a copper smelting plant in Chuquicamata, Chile. Mean values of exposure periods and total arsenic concentration in the urine of the workers were 15.55 years and 152.8 ppb, respectively. In this study, nine polymorphisms were found from the sequencing of exons and flanking regions of *AS3MT* gene. Three polymorphisms, *AS3MT* 4965 (rs17881215), exon 1: 5′-UTR (inside VNTR), and 14458 (rs11191439) in *AS3MT*, which comprised one LD cluster, were significantly associated with the variation in %MMA[V].

To confirm the genetic polymorphism-dependent metabolism of arsenic, Hernández *et al.* [45] re-investigated the relationships of the arsenic profile and *AS3MT* SNPs in larger population (*n* = 207), in which the urine had 91.26 ppb of the arsenic level on average. The results revealed that exonic *AS3MT* 14458 (rs11191439) TC + CC showed higher %MMA[V] in the urine than the wild type as observed in the previous study [44]. In summary, the authors concluded that *AS3MT* 14458 (rs11191439) TC + CC genotypes increase the methylation capacity from IA to MMA.

#### 3.2.4. Central Europe

Lindberg *et al.* [46] investigated the association between the genetic polymorphism in *AS3MT* and arsenic metabolism in populations from Hungary, Romania and Slovakia (*n* = 415) that have been exposed to low arsenic levels. This investigation is distinct from others, and is meaningful because variation of the arsenic metabolism depending on the exposure could be negligible. Even though the median urinary arsenic concentration of the individuals was low (8.0 μg/L), percentages of arsenic compounds in the urine varied widely. Through the multivariate analysis, they found that *AS3MT* 14458 (rs11191439) in exon was one of the major factors that can influence arsenic metabolism; the TC + CC type had higher %MMA[V] and lower %DMA[V] than the TT type. This trend was much more pronounced in men than in women.

#### 3.2.5. India

West Bengal in India is one of the most severe arsenic endemic areas in the world. It has been estimated that about 6,000,000 people are exposed to arsenic through the consumption of groundwater [3]. However, only 300,000 individuals (5% of total exposed population) within this population had arsenic-related disease [47]. De Chaudhuri *et al.* [48] hypothesized that low occurrence of the disease may be related to the genetic variability. Hence, the association of exonic genetic polymorphisms in *AS3MT* with the occurrence of arsenic-induced skin lesions was investigated. They first analyzed 13 genotypes in *AS3MT* in 25 cases with skin lesions and 25 controls from North 24 Parganas, Nadia, and Murshidabad Districts in West Bengal, and identified eight common genotypes. Mean concentrations of arsenic in drinking water and human urine were 163.16 μg/L and 301.17 μg/L, respectively, in the cases with skin lesions, and 161.71 μg/L and 283.28 μg/L, respectively, in controls. For these samples, there were no significant differences in the arsenic concentrations between case and control groups. On the other hand, arsenic concentrations in nail and hair of case subjects (5.39 μg/g dry wt and 3.13 μg/g dry wt, respectively) were significantly higher than those of control group (2.39 μg/g dry wt and 1.61 μg/g dry wt, respectively). The authors did not carry out urinary arsenic speciation and thus they did not mention arsenic methylation. Among the identified eight genotypes, *AS3MT* 14458 (rs11191439) was genotyped in 229 cases and 199 controls, and the distribution of genotype frequencies was compared between cases and controls. However, no significant difference was detected between the two groups, and thus the exonic genetic polymorphisms in *AS3MT* may not be linked to the occurrence of arsenic-induced skin lesions.

#### 3.2.6. Taiwan

Chung *et al.* [49] attempted to evaluate the impact of *AS3MT* 12390 (rs3740393) in intron 6 on the cancer prevalence in arsenic-endemic areas of Taiwan. The authors conducted a 15 years follow-up study (1988–2004) in Homei, Funshin, and Hsinming villages in Putai Township of Chiayi County, where the highest prevalence of Black Footed Disease has been observed. During these 15 years, total arsenic concentration, %IA, and %MMA[V] significantly decreased, while contrasting results were observed for %DMA[V], (MMA[V] + DMA[V])/total arsenic, and DMA[V]/MMA[V]. Individuals with higher %MMA[V] in 1988 and with little change in %MMA[V] during these 15 years had higher cancer risk, but its significance level was low (*p* < 0.1). These results imply that %MMA[V] is a potential predictor of the cancer risk. Seventeen cases with cancers identified by the National Cancer Registry Systems and 191 controls were investigated, but *AS3MT* 12390 (rs3740393) showed no significant effect on the cancer risk. On the other hand, a general linear model (GLM), which was adjusted for age, sex, and education level, revealed that *AS3MT* 12390 (rs3740393) GC genotype had lower %MMA[V] and higher %DMA[V] and DMA[V]/MMA[V] than the GG genotype. This suggests that the GC carrier may have a higher methylation capacity from MMA to DMA and this SNP may be an indicator of individual cancer susceptibility in relation to MMA%.

#### 3.2.7. Vietnam

Arsenic pollution in groundwater in Vietnam was reported by Berg *et al* [50]. The authors detected more than 3,000 μg/L of As concentration in groundwater from around Hanoi. Following this initial attempt, many studies have reported the concentration and distribution patterns of As in groundwater and sediment samples in northern and southern Vietnam [51–56].

While intensive studies have focused on the levels of arsenic in environmental samples, there is little information on arsenic exposure to, and toxic impacts on, local residents [18,53,57–61]. Furthermore, frequency distribution of *AS3MT* polymorphisms and the association with arsenic methylation ability have not yet been clarified. Hence, our group has investigated arsenic exposure, methylation capacity, and SNPs in *AS3MT* in Vietnamese [17–21].

To clarify the relationship between arsenic methylation and SNPs in *AS3MT*, we collected groundwater as well as human hair, urine, and blood samples from Hoa Hau and Liem Thuan in Ha Nam Province, which are located in the Red River Delta. For the human samples, each arsenic compound was quantified and 17 SNPs in *AS3MT* were genotyped [17,18,20]. The arsenic level in filtered water that some families consumed in Hoa Hau drastically (around 93%) decreased when compared with that in raw groundwater. Geometric means (GMs) of arsenic concentration in drinking water (50.1 μg/L) and human hair (0.351 μg/g dry wt) from Hoa Hau were significantly higher than those from Liem Thuan (1.7 μg/L in the water and 0.232 μg/g dry wt in the hair). On the other hand, there was no significant difference in urinary arsenic level and composition of arsenicals between Hoa Hau (GM, 92.6 μg/g creatinine) and Liem Thuan (GM, 97.9 μg/g creatinine), suggesting that the residents in Hoa Hau have not recently been exposed to arsenic through drinking water and/or there are arsenic sources other than drinking water in Liem Thuan. Among the 17 SNPs in *AS3MT* investigated, 14 unexonic SNPs were categorized into four LD clusters; *AS3MT* 3963 (rs7098825), 6144 (rs17878846), 12390 (rs3740393), 14215 (rs3740390), 35587 (rs11191453), and 37950 (rs17879819) as cluster 1, *AS3MT* 4602 (rs7085104), 35991 (rs10748835), and 37853 (rs11191459) as cluster 2, *AS3MT* 4740 (rs12416687) and 12590 (rs3740392) as cluster 3, and *AS3MT* 5913 (rs4917986), 9749 (rs17881367), and 27215 (rs11191446) as cluster 4. We also found that sex, age, and body mass index (BMI) are correlated with urinary arsenic profile in this Vietnamese population. Thus, to understand the SNP-dependent association with arsenic metabolism without co-effects, we further conducted an analysis that was adjusted for sex, age, and BMI and detected significant associations for 12 SNPs. In cluster 1, homozygotes in *AS3MT* 12390 (rs3740393) GG and 35587 (rs11191453) CC had lower DMA[V]/MMA[V] in the urine, suggesting low methylation capacity from MMA to DMA for homo types of these SNPs. All homozygotes for *AS3MT* 4602 (rs7085104) GG, 35991 (rs10748835) AA, and 37853 (rs11191459) AA in cluster 2 had lower %DMA[V] in the urine, when compared with other genotypes in the corresponding SNP. For *AS3MT* 37853 (rs11191459), %MMA[V] in AA homozygote was lower than in GG type. LD cluster 3 (*AS3MT* 4740 (rs12416687) and 12590 (rs3740392)) showed significant associations with urinary %DMA[V]. For *AS3MT* 12590 (rs3740392), the TT type had a higher DMA[V]/MMA[V] ratio in urine than other genotypes, suggesting the prompted second methylation capacity. The LD group including *AS3MT* 5913 (rs4917986), 9749 (rs17881367), and 27215 (rs11191446) (cluster 4) had a correlation with %MMA[V]. For *AS3MT* 5913 (rs4917986), TC type had a higher MMA[V]/IAs than TT type, suggesting that the SNP may be related to the first methylation process of As. *AS3MT* 27215 (rs11191446) AA had a higher DMA[V]/MMA[V] and a lower MMA[V]/IA than SNP27215 (rs11191446) AG. Apart from LD clusters, *AS3MT* 8979 (rs7920657) AA showed a low %DMA[V] compared with other genotypes. Heterozygote for exonic *AS3MT* 14458 (rs11191439, Met287Thr) TC had a higher MMA[V]/IAs in urine than TT homozygote, indicating that the heterozygote may have a stronger methylation ability of IAs.

## 4. Allele Frequency of SNPs in *AS3MT*

As we have mentioned above, several SNPs in *AS3MT* may influence arsenic metabolism. However, only limited information on frequencies of genetic polymorphisms in *AS3MT* was available for the number of the SNPs and the association with ethnicity. We have reported distribution of SNPs in *AS3MT* in some Asian [Japanese (JP), South Koreans (SKR), Chinese (CH), Mongolians (MN), Tibetans (TIB), Nepalese (NP), Vietnamese (VN), Sri Lanka-Tamils (SLT), Sri Lanka-Sinhalese (SLS)] and African [Ovambos (OVA) and Ghanaians (GH)] populations since 2007 [18,20,62–65].

**Figure 3.**
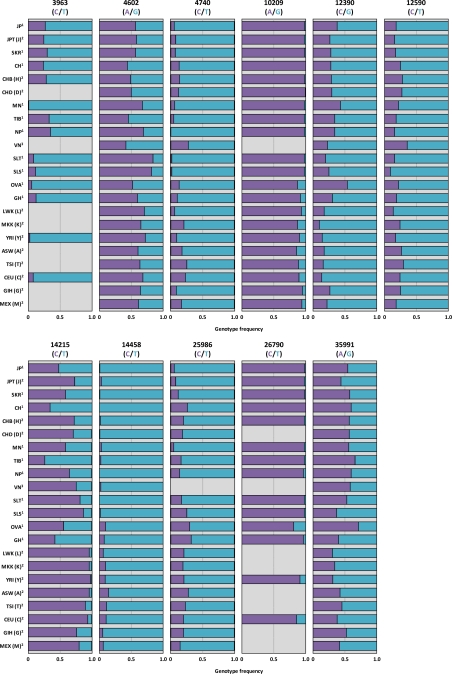
Frequencies of alleles in *AS3MT* in various populations reported in our studies [20,65] and the HapMap Project [31]. JP: Japanese, JPT (J): Japanese in Tokyo, Japan, SKR: South Koreans, CH: Chinese, CHB (H): Han Chinese in Beijing, China, CHD (D): Chinese in Metropolitan Denver, Colorado, MN: Mongolians, TIB: Tibetans, NP: Nepalese, VN: Vietnamese, SLT: Sri Lanka-Tamils, SLS: Sri Lanka-Sinhalese, OVA: Ovambos, GH: Ghanaians, LWK (L): Luhya in Webuye, Kenya, MKK (K): Maasai in Kinyawa, Kenya, YRI (Y): Yoruban in Ibadan, Nigeria, ASW (A): African ancestry in Southwest USA, TSI (T): Tuscan in Italy, CEU (C): Utah residents with Northern and Western European ancestry from the CEPH collection, GIH (G): Gujarati Indians in Houston, Texas, MEX (M): Mexican ancestry in Los Angeles, California. 1; Fujihara *et al.* [65], 2; Agusa *et al.* [20], 3; HapMap Project [31].

The summarized results are shown in Table 1. The genotype frequencies followed the Hardy–Weinberg Principle except for 4602 (rs7085104) in MN, 6144 (rs17878846) in CH and TIB, 7395 (rs12767543) in SKR and VN, 9749 (rs17881367) in MN, 12390 (rs3740393) in JP, 12590 (rs3740392) in JP, 14215 (rs3740390) in JP, SKR, MN, and GH, 26790 (rs11191445) in JP, 35587 (rs11191453) in JP, SKR, MN, TIB, and VN, 37616 (rs4568943) in JP, CH, Mongolians, and OVA, and 37950 (rs17879819) in OVA. Because no genotyping error was observed in duplicate analysis, the reason that some genotype frequencies did not follow the Hardy–Weinberg Principle may probably be due to the small sample size. The allele frequencies of SNPs of Japanese in Tokyo, Japan (JPT (J)), Han Chinese in Beijing, China (CHB (H)), Chinese in Metropolitan Denver, Colorado (CHD (D)), Luhya in Webuye, Kenya (LWK (L)), Maasai in Kinyawa, Kenya (MKK (K)), Yoruban in Ibadan, Nigeria (YRI (Y)), African ancestry in Southwest USA (ASW (A)), Tuscan in Italy (TSI (T)), Utah residents with Northern and Western European ancestries from the CEPH collection (CEU (C)), Gujarati Indians in Houston, Texas (GIH (G)), and Mexican ancestry in Los Angeles, California (MEX (M)) that are available in the International HapMap Project [31], were compared with our studies (Figure 3). While the distribution patterns of *AS3MT* 4602 (rs7085104), 9749 (rs17881367), 12390 (rs3740393), 12590 (rs3740392), and 27215 (rs11191446) polymorphisms were not drastically different among the populations, notable variations were found for other SNPs. For *AS3MT* 3963 (rs7098825), C alleles were low in SLT, SLS, OVA, GH, YRI (Y) and CEU (C), particularly in MN. NP has no C allele of *AS3MT* 4740 (rs12416687). Relatively high frequencies of *AS3MT* 7395 (rs12767543) A allele and 8979 (rs72920657) A allele were found in CH and VN, respectively. *AS3MT* 6144 (rs17878846) A allele, *AS3MT* 37616 (rs4568943) A allele, and *AS3MT* 37950 (rs17879819) C allele frequencies in OVA and GHA populations were higher than Asians, but comparable with those in MN. Frequencies of *AS3MT* 10209 (rs3740394) A allele, *AS3MT* 26790 (rs11191445) C allele, and *AS3MT* 35991 (rs10748835) A allele in Asians (except for *AS3MT* 35991 (rs10748835) A allele in OVA) were higher than those in other ethnic groups, while the opposite trend was observed for *AS3MT* (rs11191439) T allele. For *AS3MT* 14215 (rs3740390), the allele distribution was different even within Chinese (CH *vs* CHB(H) and CHD(D)) and African groups (OVA and GH *vs* LWK(L), MKK(K), YRI(Y), and ASW(A)). TIB also showed a different pattern of *AS3MT* 14215 (rs3740390) allele from other Asian populations. Relatively lower C allele frequency of *AS3MT* 25986 (rs7085854) was detected in JP, JPT, SKR, and MN. TIB showed different allele distribution of *AS3MT* 35587 (rs11191446). To understand these variations, further research is necessary.

## 5. Conclusions and Future Perspectives

**Figure 4.**
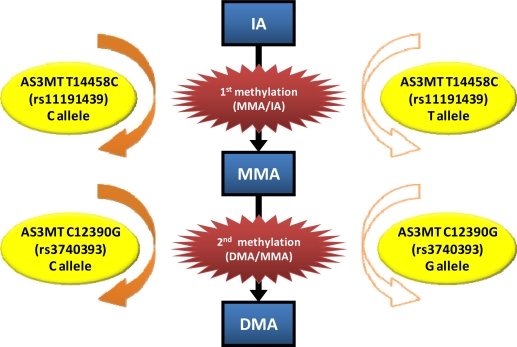
Suspected universal *AS3MT* genotype-dependent methylation of arsenic. C carrier of *AS3MT* 14458 (rs11191439) may have higher first methylation capacity than the T carrier, while C carrier of *AS3MT* 12390 (rs3740393) may have higher second methylation capacity than the G carrier.

In this review, we summarized recent results on association of *AS3MT* genetic polymorphism with arsenic methylation. Although there are some inconsistencies between the genotypes and metabolism in human case studies, we found consistent results on two SNPs, *AS3MT* 12390 (rs3740393) in intron and 14458 (rs11191439, Met287Thr) in exon, in all the nations (Figure 4 and Table 2). This indicates that these SNPs may be ethnically independent polymorphisms, but may affect arsenic methylation.

*AS3MT* 12390 (rs3740393) is an intronic polymorphism, but the genotype with C allele in this SNP may have a higher second methylation capacity as predicted from the consistent results of high DMA[V]/MMA[V] in Mexicans, Argentines, Taiwanese, and Vietnamese (Table 2). Although *AS3MT* 12390 (rs3740393) belongs to a LD cluster in these populations, other SNPs within the cluster are different among populations (Table 2). Therefore, *AS3MT* 12390 (rs3740393) may be involved in an independent reaction from other SNPs in the same LD on a worldwide population basis. The frequency of *AS3MT* 12390 (rs3740393) CC or CG types was much higher than the GG type in Argentina’s population; the allele frequencies of C and G were 72% and 28%, respectively [40]. This allele frequency distribution was drastically different from other populations (Table 1 and Figure 3). This unique distribution may contribute to the results indicating that Argentina’s population has a higher %DMA and a lower %MMA in the urine, when compared with yet another study [15]. It is noteworthy to study whether this specific genotype distribution of *AS3MT* 12390 (rs3740393) is observed only in this group (Argentinean Andes) and how this unique SNP selection took place.

*AS3MT* 14458 (rs11191439), locates in the exon region, is synonymous substitution (Met to Thr substitution) at the amino acid base 287, and its functional difference has been investigated in some studies. By reviewing the *in vitro* [30,34] and human case studies [17–21,35–40,43–49], we found that C allele containing genotype in this SNP consistently showed a higher first methylation capacity than the T allele carrier. Interestingly, the effect of this SNP has been observed even in subjects exposed to low levels of arsenic [46]. Hence, it seems that the association of *AS3MT* 14458 polymorphism with arsenic methylation may occur irrespective of arsenic exposure status. This allele frequency was relatively low in Asian populations (Table 1 and Figure 3), indicating that among different global populations, Asians may be genetically less sensitive to arsenic methylation.

Increased %MMA in the urine linked to cancer risk has been suggested in previous epidemiological studies [9–12]. Thus, *AS3MT* 14458 (rs11191439) C allele carriers, who have a higher methylation capacity from IA to MMA, may be at higher risk of arsenic related cancers. The association of this SNP with human health effect was investigated in Mexicans [37] and Indians [48]. According to the study by De Chaudhuri *et al.* [48], there was no significant interaction between this SNP and skin lesions. On the other hand, Valenzuela *et al.* [37] found higher frequency of C allele type of *AS3MT* 14458 (rs11191439) TC + CC in people with cancers, although the level of significance was not high (*p* = 0.055). Hence, at present, no rigid evidence has been provided to link the *AS3MT* 14458 (rs11191439) dependent variation in arsenic methylation to arsenic-induced carcinogenesis. Apart from an evidence of cancer, Sampayo-Reyes *et al.* [39] found that *AS3MT* 14458 (rs11191439) is linked with DNA damages among children living in arsenic contaminated sites in Mexico (*p* = 0.034). Although these authors did not analyze arsenic compounds in urine and thus did not take into consideration the methylation capacity, they suggested that increased production of MMA[III] during methylation processes in *AS3MT* 14458 (rs11191439, Met287Thr) C allele carrier might induce genotoxicity.

Up to now, it still remains unknown how *AS3MT* 12390 (rs3740393) and 14458 (rs11191439) can influence the structure and function of this enzyme. Also, several results of *AS3MT* genotype-dependent arsenic methylation are interesting, because some non-exonic (intron, UTR, or 5′ and 3′ gene) SNPs may play important roles during methylation. The study on splicing variants of *AS3MT* gene and their function is one such issue to be pursued.

There are several tasks for future study regarding the role of *AS3MT* polymorphisms on arsenic methylation. As shown in this review, most studies encompassed only a small number of subjects and SNPs in *AS3MT* and thus, they merely provide fragmental and weak significant results at particular level(s). As shown in Table 2, results on the genotype association of *AS3MT* 4602 (rs7085104), 5194 (rs3740400), 7395 (rs12767543), 12590 (rs3740393), 14215 (rs3740390), 35587 (rs11191453), and 35991 (rs10748835) with arsenic metabolism were not consistent among different country groups. Larger scale studies would be required to gain enough statistical power and strengthen currently proposed assumptions on the linkages of *AS3MT* SNPs with arsenic methylation and cancer risk.

SNPs in *AS3MT* can provide useful information on the medical treatment of some diseases or risk assessment. For example, arsenic trioxide has been used for the treatment of acute promyelocytic leukemia, although the systematic mechanism is not fully understood [66]. The information on gene-arsenic interactions presented here can be applied to a personalized medicine for leukemia or other cancers. To better understand the variations in arsenic metabolism and to assess the potential susceptibility to its toxic and carcinogenic effects at individual and population levels, more comprehensive studies would be necessary.

## Figures and Tables

**Table 1.** Allele frequency of genotypes in *AS3MT*of various Asians and Africans [18,20,62–65].

**SNP ID**	**rs #**	**Location**	**Nucleotide change**	**Ancestral**	**Allele**	**Japanese (*n*= 141)**	**South Koreans (*n*= 230)**	**Chinese (*n*= 54)**	**Mongolians (*n*= 58)**	**Tibetans (*n*= 65)**	**Nepalese (*n*= 31)**
3963	rs7098825	5′ terminal	C/T	T	C	0.266	0.298	0.241	0.009	0.331	0.355
					T	0.734	0.702	0.759	0.991	0.669	0.645
4602	rs7085104	5′ terminal	A/G	A	A	0.567	0.565	0.436	0.672*	0.454	0.694
					G	0.433	0.435	0.565	0.327*	0.546	0.307
4740	rs12416687	5′ terminal	C/T	T	C	0.060	0.067	0.130	0.061	0.054	0.000
					T	0.940	0.933	0.871	0.940	0.946	1.000
5913	rs4917986	Intron 3	C/T	T	C	NA	NA	NA	NA	NA	NA
					T	NA	NA	NA	NA	NA	NA
6144	rs17878846	Intron 3	A/T	A	A	0.734	0.730	0.695*	0.931	0.708*	0.678
					T	0.266	0.270	0.306*	0.069	0.292*	0.323
7395	rs12767543	Intron 3	A/G	G	A	0.287	0.274*	0.426	0.069	0.185	0.275
					G	0.713	0.727*	0.574	0.931	0.815	0.726
8979	rs7920657	Intron 5	A/T	T	A	0.057	0.076	0	0.077	0.069	0.000
					T	0.944	0.924	1.000	0.922	0.931	1.000
9749	rs17881367	Intron 5	A/G	A	A	0.944	0.924	1.000	0.922*	0.931	1.000
					G	0.057	0.076	0	0.077*	0.069	0.000
10209	rs3740394	Intron 6	A/G	T	A	0.990	0.985	1.000	0.983	0.985	0.984
					G	0.011	0.015	0	0.017	0.016	0.016
12390	rs3740393	Intron 6	C/G	C	C	0.373*	0.279	0.278	0.431	0.338	0.339
					G	0.628*	0.722	0.722	0.569	0.662	0.661
12590	rs3740392	Intron 7	C/T	T	C	0.178*	0.172	0.251	0.215	0.185	0.162
					T	0.823*	0.829	0.751	0.784	0.816	0.839
14215	rs3740390	Intron 8	C/T	C	C	0.483*	0.587*	0.343	0.587*	0.262	0.645
					T	0.518*	0.413*	0.658	0.414*	0.739	0.355
14458^1^	rs11191439	Exon 9	C/T	T	C	0.004	0.009	0	0.026	0.023	0.000
					T	0.997	0.992	1.000	0.974	0.977	1.000
25986	rs7085854	Intron 8	C/T	T	C	0.057	0.122	0.259	0.052	0.162	0.145
					T	0.944	0.879	0.741	0.949	0.839	0.855
26790	rs11191445	Intron 10	C/T	C	C	0.990*	0.985	1.000	0.983	0.977	0.968
					T	0.011*	0.015	0	0.017	0.023	0.033
27215	rs11191446	Intron 10	A/G	A	A	0.926	0.918	0.972	0.949	0.900	0.952
					G	0.075	0.083	0.028	0.052	0.100	0.049
35587	rs11191453	Intron 10	C/T	T	C	0.344*	0.370*	0.333	0.155*	0.516*	0.404
					T	0.656*	0.631*	0.666	0.845*	0.485*	0.597
35991	rs10748835	Intron 10	A/G	A	A	0.543	0.570	0.602	0.561	0.654	0.597
					G	0.457	0.431	0.398	0.440	0.346	0.404
37616	rs4568943	3′ terminal	A/C	A	A	0.656*	0.735	0.519*	0.906*	0.639	0.678
					C	0.344*	0.266	0.482*	0.095*	0.362	0.323
37853	rs11191459	3′ terminal	A/G	G	A	NA	NA	NA	NA	NA	NA
					G	NA	NA	NA	NA	NA	NA
37950	rs17879819	3′ terminal	C/T	C	C	0.710	0.713	0.676	0.923	0.638	0.629
					T	0.291	0.287	0.324	0.078	0.361	0.371
References						[65]	[65]	[65]	[65]	[65]	[65]

**SNP ID**	**rs #**	**Location**	**Nucleotide change**	**Ancestral**	**Allele**	**Vietnamese (*n*= 100)**	**Sri Lanka-Tamils (*n*= 29)**	**Sri Lanka-Sinhalese (*n*= 30)**	**Ovambos (*n*= 102)**	**Ghanaians (*n*= 87)**
3963	rs7098825	5′ terminal	C/T	T	C	NA	0.086	0.117	0.054	0.121
					T	NA	0.914	0.883	0.946	0.879
4602	rs7085104	5′ terminal	A/G	A	A	0.420	0.845	0.817	0.525	0.598
					G	0.580	0.155	0.184	0.475	0.403
4740	rs12416687	5′ terminal	C/T	T	C	0	0.017	0.017	0.133	0.104
					T	0.720	0.983	0.984	0.868	0.897
5913	rs4917986	Intron 3	C/T	T	C	0.080	NA	NA	NA	NA
					T	0.920	NA	NA	NA	NA
6144	rs17878846	Intron 3	A/T	A	A	0.750	0.845	0.867	1.000	1.000
					T	0.250	0.155	0.134	0	0
7395	rs12767543	Intron 3	A/G	G	A	0.335*	0.121	0.167	0.113	0.304
					G	0.665*	0.880	0.834	0.888	0.695
8979	rs7920657	Intron 5	A/T	T	A	0	0.035	0.017	0.142	0.075
					T	0.590	0.966	0.984	0.858	0.926
9749	rs17881367	Intron 5	A/G	A	A	NA	0.966	0.984	0.858	0.926
					G	NA	0.035	0.017	0.142	0.075
10209	rs3740394	Intron 6	A/G	T	A	NA	0.983	0.984	0.868	0.920
					G	NA	0.017	0.017	0.133	0.081
12390	rs3740393	Intron 6	C/G	C	C	0.225	0.189	0.251	0.540	0.299
					G	0.775	0.810	0.751	0.461	0.702
12590	rs3740392	Intron 7	C/T	T	C	0.360	0.155	0.084	0.221	0.189
					T	0.640	0.845	0.917	0.780	0.810
14215	rs3740390	Intron 8	C/T	C	C	0.755	0.810	0.867	0.554	0.414*
					T	0.245	0.189	0.134	0.446	0.587*
14458^1^	rs11191439	Exon 9	C/T	T	C	0	0	0.017	0.093	0.075
					T	0.980	1.000	0.984	0.907	0.926
25986	rs7085854	Intron 8	C/T	T	C	NA	0.172	0.250	0.299	0.316
					T	NA	0.828	0.750	0.701	0.684
26790	rs11191445	Intron 10	C/T	C	C	NA	0.983	0.984	0.814	0.966
					T	NA	0.017	0.017	0.186	0.035
27215	rs11191446	Intron 10	A/G	A	A	NA	0.966	0.984	0.873	0.920
					G	NA	0.035	0.017	0.128	0.081
35587	rs11191453	Intron 10	C/T	T	C	0.270*	0.207	0.134	0.045	0.063
					T	0.730*	0.793	0.867	0.957	0.937
35991	rs10748835	Intron 10	A/G	A	A	0.585	0.535	0.367	0.706	0.402
					G	0.415	0.466	0.633	0.294	0.598
37616	rs4568943	3′ terminal	A/C	A	A	NA	0.794	0.867	0.941*	0.931
					C	NA	0.207	0.134	0.059*	0.069
37853	rs11191459	3′ terminal	A/G	G	A	0.495	NA	NA	NA	NA
					G	0.505	NA	NA	NA	NA
37950	rs17879819	3′ terminal	C/T	C	C	NA	0.793	0.867	0.961*	0.937
					T	NA	0.207	0.134	0.040*	0.063
References						[20]	[65]	[65]	[65]	[65]

NA: no available data.

*: not followed Hardy–Weinberg Principle (*p* < 0.05).

1: Met to Thr substitution at amino acid base on 287.

**Table 2.** Differences in the association of arsenic methylation and linkage disequilibrium (LD) for SNPs of *AS3MT* among nations.

**SNP ID**	**rs #**	**Location**	**Nucleotide change**	**Ancestral**	**Mexico (*n* = 144)**	**Mexico (*n* = 122)**	**Mexico (*n* = 405)**	**Argentina (*n* = 147)**
3963	rs7098825	5′ terminal	C/T	T				
4602	rs7085104	5′ terminal	A/G	A	NS	%MMA[III + V]: AA + AG > GG %DMA[III + V]: GG > AA + AG DMA[III + V]/MMA[III + V]: GG > AA + AG		
4740	rs12416687	5′ terminal	C/T	T	NS			
4965	rs17881215	Exon 1: 5′ UTR	C/G	G				
5019	rs17880342	Exon 1: 5′ UTR	C/T	C				NA
5051	rs17883409	Intron 1	*	NAV	NS			NA
5194	rs3740400	Intron 1	A/C	C				NA
5913	rs4917986	Intron 3	C/T	T				
6144	rs17878846	Intron 3	A/T	A				
7395	rs12767543	Intron 3	A/G	G	DMA[V]/MMA[V]: AA + AG > GG^2^		S	
8788	rs17881367	Intron 5	-/TTT	NAV				NA
8979	rs7920657	Intron 5	A/T	T				
9749	rs17881367	Intron 5	A/G	A				
10209	rs3740394	Intron 6	C/T	T	NS			
12390	rs3740393	Intron 6	C/G	C	DMA[V]/MMA[V]: CC + CG > GG^2^		S	%MMA[V]: GG > CC, CG %DMA[V]: CC, CG > GG DMA[V]/MMA[V]: CC, CG > GG
12590	rs3740392	Intron 7	C/T	T	NS			NA
13599	rs10883797	Intron 7	C/G	C	NS			
14215	rs3740390	Intron 8	C/T	C			S	%MMA[V]: CC > TT, CT %DMA[V]: TT, CT > CC DMA[V]/MMA[V]: TT, CT > CC
14458^1^	rs11191439	Exon 9	C/T	T		%MMA[III + V]: CT + CC > TT %As[V]: CT + CC > TT	NS	NA
25986	rs7085854	Intron 9	C/T	T	NS			
27215	rs11191446	Intron 10	A/G	A				
30312	rs1191453	Intron 10	C/T	T			DMA[V]/MMA[V]: CC > CT > TT	
35587	rs11191453	Intron 10	C/T	T	DMA[V]/MMA[V]: CC + CT > TT^2^ MMA[V]/As[III]: TT > CC + CT^2^	NS	S	
35739	rs11191454	Intron 10	A/G	A			S	
35991	rs10748835	Intron 10	A/G	A				%MMA[V]: GG > AA, AG %DMA[V]: AA, AG > GG DMA[V]/MMA[V]: AA, AG > GG
36209	rs17879039	Exon 11: 3′ UTR	A/G	G				
37219	rs1046778	Exon 11: 3′ UTR	C/T	T			S	
37616	rs4568943	3′ terminal	A/C	A				
37853	rs11191459	3′ terminal	A/G	G				
37950	rs17879819	3′ terminal	C/T	C				
LD cluster								
LD1					7395-12390-35587		7395-12390-14215-30312 -35587-35739-37219	12390-14215-35991
LD2								
LD3								
LD4								
References					[35, 36]	[37]	[38]	[40]

**SNP ID**	**rs #**	**Location**	**Nucleotide change**	**Ancestral**	**Argentina (*n* = 104)**	**Chile (*n* = 50)**	**Chile (*n* = 207)**
3963	rs7098825	5′ terminal	C/T	T			
4602	rs7085104	5′ terminal	A/G	A	%MMA[V]: AA + AG > GG %DMA[V]: GG > AG + AA		
4740	rs12416687	5′ terminal	C/T	T			
4965	rs17881215	Exon 1: 5′ UTR	C/G	G		%MMA: CG > GG	
5019	rs17880342	Exon 1: 5′ UTR	C/T	C			
5051	rs17883409	Intron 1	*	NAV			
5194	rs3740400	Intron 1	A/C	C	%MMA[V]: AA + AC > CC %DMA[V]: CC > AC + AA	NS	
5913	rs4917986	Intron 3	C/T	T			
6144	rs17878846	Intron 3	A/T	A			
7395	rs12767543	Intron 3	A/G	G			
8788	rs17881367	Intron 5	-/TTT	NAV			
8979	rs7920657	Intron 5	A/T	T			
9749	rs17881367	Intron 5	A/G	A			
10209	rs3740394	Intron 6	C/T	T			
12390	rs3740393	Intron 6	C/G	C			
12590	rs3740392	Intron 7	C/T	T			
13599	rs10883797	Intron 7	C/G	C			
14215	rs3740390	Intron 8	C/T	C			
14458^1^	rs11191439	Exon 9	C/T	T		%MMA: CT + CC > TT	%MMA: CT + CC > TT
25986	rs7085854	Intron 9	C/T	T			
27215	rs11191446	Intron 10	A/G	A			
30312	rs1191453	Intron 10	C/T	T			
35587	rs11191453	Intron 10	C/T	T			
35739	rs11191454	Intron 10	A/G	A			
35991	rs10748835	Intron 10	A/G	A			
36209	rs17879039	Exon 11: 3′ UTR	A/G	G		NS	
37219	rs1046778	Exon 11: 3′ UTR	C/T	T			
37616	rs4568943	3′ terminal	A/C	A			
37853	rs11191459	3′ terminal	A/G	G			
37950	rs17879819	3′ terminal	C/T	C			
LD cluster							
LD1					4602-5194-12390-14215-35991^3^	4965-14458	
LD2							
LD3							
LD4							
References					[43]	[44]	[45]

**SNP ID**	**rs #**	**Location**	**Nucleotide change**	**Ancestral**	**Central Europe^4^ (*n* = 415)**	**Taiwan (*n* = 208)**	**Vietnam (*n* = 100)**
3963	rs7098825	5′ terminal	C/T	T			NS
4602	rs7085104	5′ terminal	A/G	A			%DMA[V]: AA, AG > GG
4740	rs12416687	5′ terminal	C/T	T			%DMA[V]: TT > CT, CC
4965	rs17881215	Exon 1: 5′ UTR	C/G	G			
5019	rs17880342	Exon 1: 5′ UTR	C/T	C			
5051	rs17883409	Intron 1	*	NAV			
5194	rs3740400	Intron 1	A/C	C			
5913	rs4917986	Intron 3	C/T	T			%MMA[V]: CT > TT
6144	rs17878846	Intron 3	A/T	A			NS
7395	rs12767543	Intron 3	A/G	G			NS
8788	rs17881367	Intron 5	-/TTT	NAV			
8979	rs7920657	Intron 5	A/T	T			%DMA[V]: AT, TT > AA
9749	rs17881367	Intron 5	A/G	A			%MMA[V]: AG > AA
10209	rs3740394	Intron 6	C/T	T			
12390	rs3740393	Intron 6	C/G	C		%MMA[V]: GG > CG %DMA[V]: CG > GG DMA[V]/MMA[V]: CG > GG	%MMA[V]: GG > CC DMA[V]/MMA[V]: CG > GG
12590	rs3740392	Intron 7	C/T	T			%DMA[V]: TT > CC DMA[V]/MMA[V]: TT > CC
13599	rs10883797	Intron 7	C/G	C			
14215	rs3740390	Intron 8	C/T	C			NS
14458^1^	rs11191439	Exon 9	C/T	T	%DMA[V]: TT > CT + CC %MMA[V]: CT + CC > TT		MMA[V]/IA: CT > TT
25986	rs7085854	Intron 9	C/T	T			
27215	rs11191446	Intron 10	A/G	A			%MMA[V]: AG > AA DMA[V]/MMA[V]: AA > AG MMA[V]/IA: AG > AA
30312	rs1191453	Intron 10	C/T	T			
35587	rs11191453	Intron 10	C/T	T			DMA[V]/MMA[V]: CT > TT
35739	rs11191454	Intron 10	A/G	A			
35991	rs10748835	Intron 10	A/G	A			%DMA[V]: AG > AA
36209	rs17879039	Exon 11: 3′ UTR	A/G	G			
37219	rs1046778	Exon 11: 3′ UTR	C/T	T			
37616	rs4568943	3′ terminal	A/C	A			
37853	rs11191459	3′ terminal	A/G	G			%DMA[V]: GG, AG > AA %MMA[V]: GG > AA
37950	rs17879819	3′ terminal	C/T	C			NS
LD cluster							
LD1							3963-6144-12390-14215-35587-37950
LD2							4602-35991-37853
LD3							4740-12590
LD4							5913-9749-27215
References					[46]	[49]	[18, 20]

NAV: not available.

NS: not significant.

NA: no statistical analysis due to the small genotype frequency.

1: Met to Thr substitution at amino acid base on 287.

2: Significant result was observed for only children but not for adults.

3: Previous data of SNP is included.

4: Hungarian, Romanian, and Slovak.

*: -/CGCGCCCTGAGTCGCAGGCCGAGGAGACAGTGAGTGC

S: Significant association with DMA[V]/MMA[V] was found but no information which genotype is associated with it.
